# Real-world diagnostic value of integrating oral and ocular dryness testing in suspected Sjögren’s disease

**DOI:** 10.3389/fmed.2025.1594270

**Published:** 2025-10-15

**Authors:** Eman Seyal, Jesse Akaa, David O’Dea, Thao Nguyen, Rahmatullah W. Rahmati, Brandon M. Law

**Affiliations:** ^1^Center for Immunology and Inflammatory Diseases, Massachusetts General Hospital, Boston, MA, United States; ^2^Division of Rheumatology, Allergy, and Immunology, Massachusetts General Hospital, Harvard Medical School, Boston, MA, United States; ^3^Department of Otolaryngology–Head and Neck Surgery, Massachusetts Eye and Ear, Harvard Medical School, Boston, MA, United States

**Keywords:** Sjögren’s disease, dry eye, dry mouth, anti-Ro serology, labial salivary gland biopsy, Schirmer’s test, unstimulated whole salivary flow, sicca

## Abstract

**Background:**

Sjögren’s disease is an autoimmune condition requiring a systemic evaluation that integrates serologic, histopathologic, and glandular assessments for diagnosis. Current 2016 ACR/EULAR classification criteria includes anti-Ro serology, labial salivary gland biopsy (LSGB), and measures of oral/ocular dryness. However, oral/ocular dryness evaluations are rarely performed by rheumatologists during routine clinical care. Thus, the real-world diagnostic value of contemporaneous oral/ocular dryness testing remains poorly understood.

**Objective:**

To evaluate the added value of contemporaneous oral/ocular dryness testing (Schirmer’s test and unstimulated whole salivary flow) in meeting classification criteria for subjects evaluated with suspected Sjögren’s disease.

**Methods:**

73 subjects referred for suspected Sjögren’s disease were evaluated. Correlations between LSGB results and dryness tests, as well as LSGB results and anti-Ro serology, were evaluated. 31 subjects completed testing of oral/ocular dryness (Schirmer’s test and unstimulated whole salivary flow), anti-Ro serology, and LSGB. Diagnostic pathways were analyzed to assess the contributions of non-invasive tests (serologies and oral/ocular dryness tests) and invasive testing (LSGB) in meeting the threshold for classification criteria.

**Results:**

A significant association (*p*-value = 0.0263) was observed between LSGB positivity and positive Schirmer’s testing. No significant association was observed between LSGB positivity and anti-Ro positivity, or between LSGB positivity and low unstimulated whole salivary flow. Among those meeting classification criteria for Sjögren’s disease, 81% (30/37) met classification independently of LSGB results. Of those who completed testing, 22 met classification, among whom 68% (15/22) fulfilled criteria independently of LSGB results. Of these 15 subjects, 8 (53%) had negative LSGB with a focus score <1. While a positive LSGB was mandatory to confirm classification for seronegative subjects, only 11.8% (2/17) of anti-Ro-positive subjects required LSGB for classification.

**Conclusion:**

Objective oral/ocular dryness testing, though rarely performed in routine rheumatologic care, is a valuable complement to serology and biopsy in diagnosing Sjögren’s disease. While LSGB is essential for confirming classification in anti-Ro-negative subjects, it adds only modest value to meeting classification criteria in anti-Ro-positive subjects relative to contemporaneous glandular dryness testing. These findings support integrating objective dryness measures into routine diagnostic workflows to reduce reliance on invasive biopsies and improve diagnostic accuracy, especially in seropositive populations.

## 1 Introduction

Sjögren’s disease (SjD) is a systemic autoimmune condition causing inflammation and destruction of exocrine glands, including the salivary and lacrimal glands, resulting in the development of xerostomia and xerophthalmia in patients. SjD also commonly results in extraglandular systemic manifestations, including pulmonary, renal, neurological, or musculoskeletal involvement (1). Given its significant heterogeneity in manifestation and disease activity, diagnosing SjD remains clinically challenging, and the time elapsed between symptom development and diagnosis can span several years (2, 3).

The 2016 American College of Rheumatology/European League Against Rheumatism (ACR/EULAR) classification criteria for Sjögren’s disease were developed to standardize subject inclusion for research and clinical trials. These criteria incorporate anti-Ro serology, labial salivary gland biopsy (LSGB), and objective assessments of ocular dryness, such as Schirmer’s test (ST) or ocular staining, as well as oral dryness evaluated through unstimulated whole salivary flow (UWSF) rate (4). However, objective assessments of ocular/oral dryness are seldom utilized in routine rheumatology practice, where clinicians often rely on subjective symptom reporting or defer glandular dryness evaluations due to time constraints or lack of familiarity with standardized protocols. This highlights a critical translational gap: while the ACR/EULAR criteria emphasize rigorous, quantifiable measures for research validity, their real-world adoption remains limited, potentially hindering accurate diagnosis and patient stratification in everyday clinical care. In fact, a 2017 population-based analysis revealed that in a cohort of 106 SjD subjects, while 97 (91.5%) subjects had undergone anti-Ro serologic testing, only 9 (8.5%) underwent ST and 1 (0.95%) underwent UWSF for assessment of objective ocular and oral dryness, respectively (5). A 2023 study reports that despite its convenience, ST was performed in only 28% (20/72) of subjects, and UWSF was not performed at all (6). Both studies report a reduction in the number of subjects meeting the threshold for classification criteria, not due to negative test results, but because objective dryness testing was often not performed (5, 6). Given this significant underutilization of oral/ocular dryness testing, its clinical value in the diagnosis and evaluation of SjD, especially in comparison to that of LSGB, remains poorly understood. This prompted us to retrospectively analyze the various diagnostic pathways of subjects in our cohort to assess the contributions of non-invasive tests (serologies and ocular/oral dryness testing) and invasive testing (e.g., LSGB) in meeting the threshold for the 2016 ACR/EULAR classification criteria. Our objective of this study was to investigate the added value of performing non-invasive oral/ocular dryness testing in meeting classification criteria for subjects evaluated with suspected Sjögren’s disease.

## 2 Materials and methods

### 2.1 Study participants

Human subjects research was approved by the Institutional Review Board of Mass General Brigham (MGB). Written informed consent was obtained from all subjects for the collection of clinical data prior to enrollment in the research study. Between December 2023 and January 2025, 73 subjects presented to the Sjögren’s Disease rheumatology clinic at Massachusetts General Hospital for evaluation who provided their consent for retrospective analysis of their medical charts. Subjects underwent anti-Ro serology testing, LSGB, ST, and/or UWSF rate testing. Rheumatologic evaluations and LSGB of subjects were performed by single investigators, and ST and UWSF were also performed by the investigators, demonstrating consistent clinical practice.

### 2.2 SjD classification criteria

Sjögren’s disease status was defined using the 2016 ACR/EULAR classification criteria. Subjects were evaluated for four criteria components: anti-Ro antibody positivity and LSGB with focus score (FS) ≥ 1 foci/mm^2^, each scoring three points, as well as unanesthetized ST ≤ 5 mm/5 min and UWSF rate ≤ 0.1 mL/min, each scoring one point. Ocular staining scores, scoring one point if positive, were not investigated in our study as we aimed to study contemporaneous dryness testing which can be performed within routine rheumatology clinic visits. Subjects presenting with clinical symptoms suggestive of SjD that had undergone sufficient or complete testing such that they scored ≥4 points (i.e., had at least positive serology and histopathology, positive serology and one abnormal dryness test, or positive histopathology and one abnormal dryness test) met classification criteria. Similarly, subjects that had undergone sufficient or complete testing such that they could not score ≥4 points (i.e., at least negative serology and histopathology) did not meet SjD classification criteria. Classification status was not determined for any remaining subjects with insufficient testing.

### 2.3 Focus score dependence

Our contemporaneous dryness assessments provided us the opportunity to categorize subjects who met SjD classification criteria as FS-independent or FS-dependent. We defined FS-independent subjects as those who obtained the minimum of 4 points to meet the threshold for ACR/EULAR SjD classification criteria regardless of LSGB FS result. FS-independent subjects must be anti-Ro-positive (three points) with at least one positive oral/ocular dryness test (one to two points). Conversely, FS-dependent subjects require a LSGB FS ≥ 1 (three points) and are either seropositive (three points) with negative oral/ocular dryness testing or are seronegative with at least one positive oral/ocular dryness test (one to two points).

### 2.4 Ocular/oral dryness assessments

To perform unanesthetized ST, a Schirmer’s strip was gently inserted at the junction of the middle and outer third of the lower eyelid of the subject, ensuring it did not touch the cornea. The subject was then instructed to close their eyes gently for the duration of the test. After 5 min, the strips were removed, and the amount of wetting was recorded. A positive ST is considered ≤5 mm/5 min in at least one eye (7). To perform UWSF rate testing, the subject was asked to swallow any residual saliva in their mouth prior to starting and to not speak or make significant facial movements during the test. Saliva was collected into a pre-weighed sterile container as it pooled in the floor of the subject’s mouth. After 5 min, the collection was halted, the subject was permitted to swallow, and the collected saliva was weighed to calculate the flow rate in mL/min. A positive UWSF is considered ≤0.1 mL/min (8).

### 2.5 Clinical and serologic assessments

We obtained the following clinical and serologic data of our subjects through review of the medical charts: age, anti-Ro positivity, LSGB FS, antinuclear antibody (ANA) titer, white blood cells (WBC), hemoglobin (HGB), platelets (PLT), absolute neutrophils, absolute lymphocytes, sedimentation rate (ESR), C-reactive protein (CRP), immunoglobulin G (IgG), complement 3 (C3), complement 4 (C4), rheumatoid factor (RF), and free kappa light chain to free lambda light chain ratio (free K:L).

### 2.6 Statistical analysis

We analyzed contemporaneous glandular dryness testing in our cohort utilizing descriptive and correlative statistics. For categorical data, we performed Chi-squared or Fisher’s exact tests to determine significance in associations between variables. We analyzed the correlation between quantitative LSGB focus scores and contemporaneous dryness test (ST and UWSF) results using Spearman’s correlation. We established *p*-value < 0.05 to be statistically significant (*p < 0.05, **p < 0.01, ***p < 0.001, ****p < 0.0001). Statistical analyses were performed using Prism 10.4.0 (GraphPad Software, La Jolla, CA). Diagnostic pathways of subjects with completed serologic, histopathologic, and glandular dryness testing were visualized via a Sankey plot by organizing test data into source-target pairs with corresponding flow magnitudes. The plot was generated using SankeyMATIC software, with nodes representing outcomes of classification criteria components and link thickness made proportional to the number of subjects in each category.

## 3 Results

**FIGURE 1:**
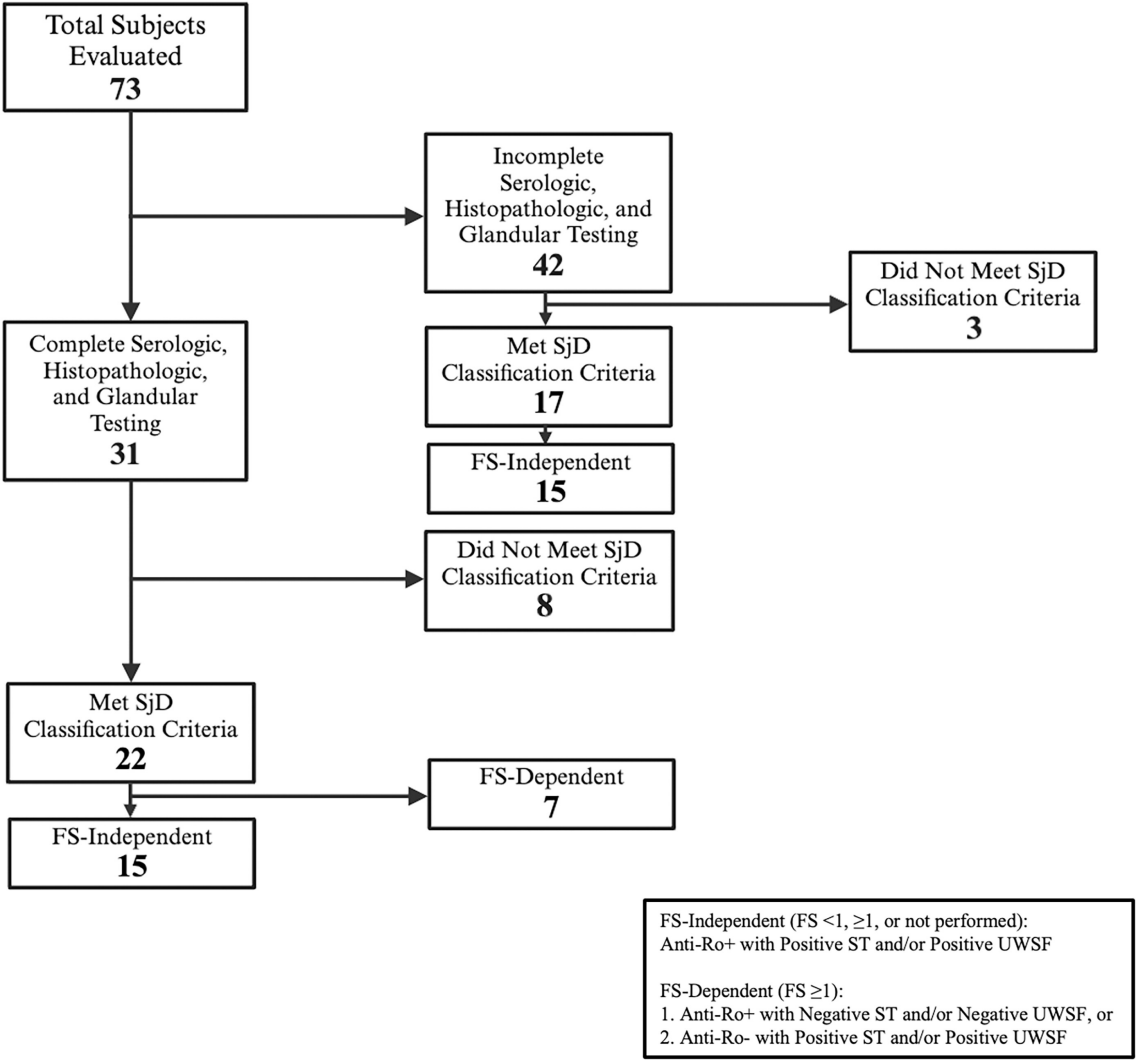
Flowchart of subjects evaluated for Sjögren’s Disease. Representation of subjects who underwent testing for either all or some components of the 2016 ACR/EULAR SjD classification criteria. Classification status is determined for all subjects with sufficient testing. FS-independence or FS-dependence is determined for all subjects who met SjD classification criteria and completed sufficient testing to be categorized.

Among all subjects with complete serologic, histopathologic, and oral/ocular dryness assessments, 22 met the 2016 ACR/EULAR SjD classification criteria threshold while 8 did not. Among all subjects with incomplete assessments which were sufficient to determine classification status, 17 met classification criteria while 3 did not. Among the 22 subjects who met SjD classification criteria with comprehensive testing, we identified 15 to be FS-independent. Among the 17 subjects who met SjD classification criteria without comprehensive testing, we identified 15 to be FS-independent (Figure 1).

Among all subjects evaluated that did and did not meet the threshold for classification criteria, we analyzed the percentage of subjects with abnormal serologic results. We found significant associations between meeting classification criteria and exhibiting positive anti-Ro antibodies (*p*-value < 0.0001 by definition), positive LSGB FS (*p*-value = 0.0001), positive ST (*p*-value = 0.0285), positive RF (*p*-value = 0.0024), and elevated free K:L (*p*-value = 0.0161), respectively (Table 1).

**TABLE 1:** Demographic, clinical, and serologic data of subjects.

Clinical parameter	Meet Classification criteria N = 39 n/N (%)	Do not meet Classification criteria N = 11 n/N (%)	*P*-value
Mean age	57	54	
Female	38/39 (97.4)	11/11 (100)	1.0000
ANA ≥ 1:320	18/33 (56.3)	6/11 (54.5)	1.0000
Positive anti-Ro	34/39 (87.2)	0/11 (0)	<0.0001****
LSGB FS ≥ 1	18/26 (69.2)	0/11 (0)	0.0001***
ST ≤ 5 mm/5 min	20/38 (52.6)	1/10 (10)	0.0285*
UWSF ≤ 0.1 mL/min	25/31 (80.6)	7/8 (87.5)	1.0000
WBC < 4.5 K/uL	11/38 (28.9)	3/10 (30)	1.0000
HGB < 12.0 g/dL	15/38 (39.5)	2/10 (20)	0.4587
PLT < 150 K/uL	5/38 (13.2)	1/10 (10)	1.0000
Absolute neutrophils < 1.8 K/uL	3/38 (7.9)	2/10 (20)	0.2758
Absolute lymphocytes < 1.2 K/uL	10/38 (26.3)	3/10 (30)	1.0000
Positive ESR > 19 mm/h	11/29 (37.9)	3/11 (27.3)	0.7152
Positive CRP > 8.0 mg/L	11/33 (33.3)	4/11 (36.4)	1.0000
Total IgG > 1295 mg/dL	16/35 (45.7)	1/9 (11.1)	0.1215
C3 < 81 mg/dL	1/33 (3)	2/11 (18.2)	0.1495
C4 < 12 mg/dL	1/35 (2.9)	0/11 (0)	1.0000
Positive RF > 14 IU/mL	18/33 (54.5)	0/10 (0)	0.0024**
Free K:L > 1.7	16/34 (47)	0/9 (0)	0.0161*

Percentages of subjects with abnormal results are categorized based on meeting or not meeting the threshold for ACR/EULAR SjD classification criteria. *P*-values listed were calculated by Fisher’s test. Significant *p*-values comparing percentages are labeled as follows: *p < 0.05, **p < 0.01, ***p < 0.001, ****p < 0.0001. ANA, antinuclear antibody; WBC, white blood cells; HGB, hemoglobin; PLT, platelets; ESR, sedimentation rate; CRP, C-reactive protein; IgG, immunoglobulin G; C3, complement 3; C4, complement 4; RF, rheumatoid factor; Free K:L, free kappa light chain to free lambda light chain ratio.

**FIGURE 2:**
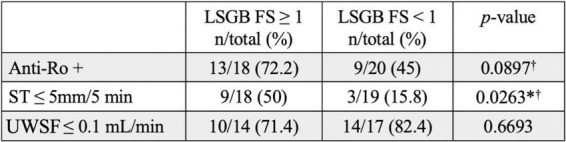
Abnormal ST is significantly associated with abnormal LSGB focus score. Percentages of subjects with positive serologic and oral/ocular dryness testing (ST and UWSF) are listed for those with positive and negative LSGB. Unless indicated (†: Chi-squared test), *P*-values were calculated by Fisher’s test. *P-*values are listed for association between LSGB FS and other components of ACR/EULAR SjD classification criteria (*p < 0.05).

We tested for the presence of any association between positive LSGB and abnormalities in other components of the classification criteria; namely anti-Ro serology, ST, and UWSF. We observed a statistically significant association between LSGB FS positivity and ST positivity (*p*-value = 0.0263, OR = 5.33, 95% CI [1.14, 24.9]). Among subjects with a positive LSGB FS, 50% (9/18) had positive ST, whereas among subjects with a negative LSGB FS, 15.8% (3/19) had positive ST. We did not observe a statistically significant association between LSGB FS positivity and either anti-Ro positivity (*p*-value = 0.0897, OR = 3.18, 95% CI [0.82, 12.33]) or UWSF rate positivity (*p*-value = 0.6693, OR = 0.54, 95% CI [0.1, 2.94]) (Figure 2).

**FIGURE 3:**
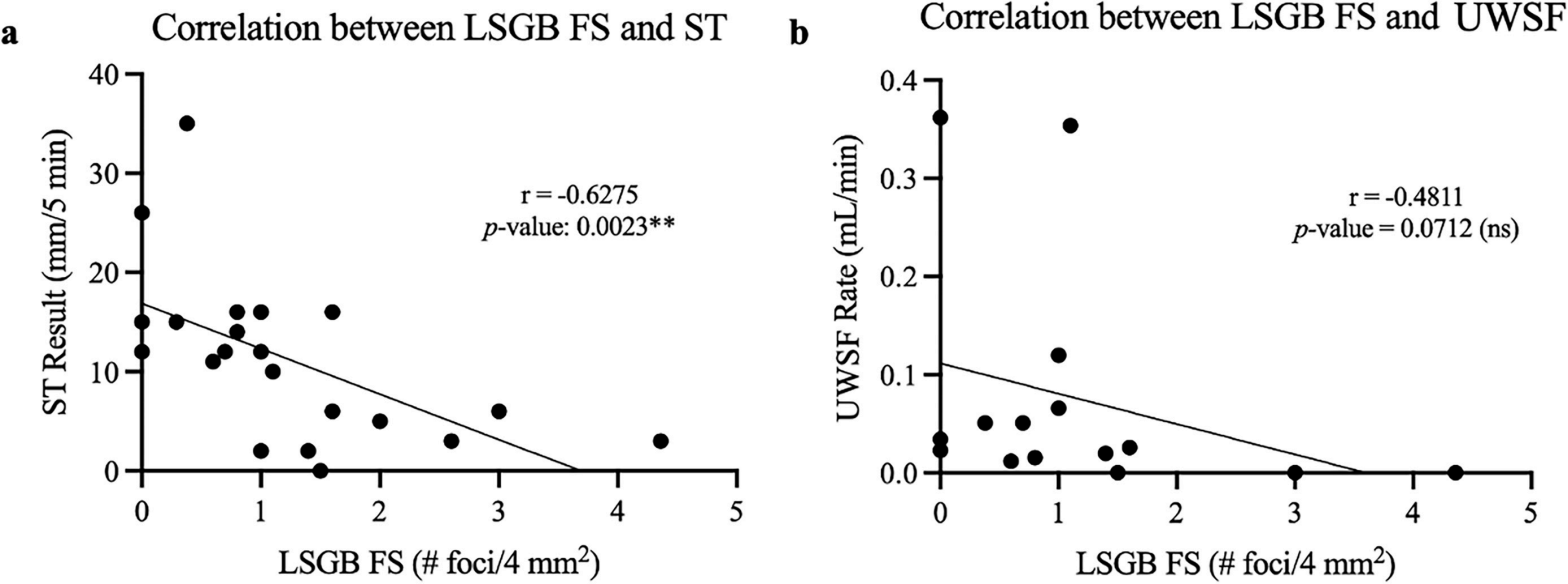
Significant negative correlation between ST and LSGB focus score. **(a)** Graph of Spearman’s correlation for subjects with ST and LSGB FS results, with correlation coefficient of –0.6275. *P*-value is listed for correlation between LSGB FS and ST (**p < 0.01). **(b)** Graph of Spearman’s correlation for subjects with UWSF and LSGB FS results, with correlation coefficient of –0.4811. *P*-value is listed for correlation between LSGB FS and UWSF rate.

Given the significant association between abnormal LSGB and ST, we then examined whether a correlation existed between the degree of inflammation observed in the LSGB FS and the degree of objective ocular and oral dryness observed in ST and UWSF. We observed that a higher FS correlates with lower ST (*p*-value = 0.0023), but, interestingly, we did not observe a significant correlation between LSGB FS and UWSF (*p*-value = 0.0712) (Figure 3).

We next decided to specifically analyze the 22 subjects that met SjD classification and underwent complete serologic, histopathologic, and oral/ocular dryness assessments to understand the added value of ST and UWSF in meeting classification criteria. Among those that met classification, 15/22 (68.2%) subjects were FS-independent and 7/22 (31.8%) subjects were FS-dependent. Among the 15 FS-independent subjects who met classification criteria, all (100%) were anti-Ro-positive (statistically significant association by definition), only 7/15 (46.7%) had positive LSGB, 8/15 (53.3%) had positive ST, and 12/15 (80%) had positive UWSF rates. Among the 7 FS-dependent subjects who met criteria, only 2/7 (28.6%) were anti-Ro-positive, all subjects (100%) had positive LSGB results (statistically significant association by definition), 1/7 (14.3%) had positive ST, and 5/7 (71.4%) had positive UWSF rates. No other statistically significant associations were observed between these two groups. 15 FS-independent subjects and 2 FS-dependent subjects were anti-Ro-positive for a total of 17 anti-Ro-positive subjects. Remarkably, anti-Ro seropositivity paired with oral/ocular dryness testing sufficed for classification in most seropositive cases, given that only 2/17 (11.8%) seropositive SjD cases were FS-dependent (Table 2).

**TABLE 2:** Demographic, clinical, and serologic data of FS-independent and FS-dependent subjects that meet SjD classification.

Clinical parameter	FS-independent N = 15 n/total (%)	FS-dependent N = 7 n/total (%)	*P*-value
Mean age	61	56	
Female	15/15 (100)	6/7 (85.7)	0.3182
ANA ≥ 1:320	6/13 (46.2)	3/7 (42.9)	0.6693
Positive anti-Ro	15/15 (100)	2/7 (28.6)	0.0008***
LSGB FS ≥ 1	7/15 (46.7)	7/7 (100)	0.0225*
ST ≤ 5 mm/5 min	8/15 (53.3)	1/7 (14.3)	0.1649
UWSF ≤ 0.1 mL/min	12/15 (80)	5/7 (71.4)	1.0000
WBC < 4.5 K/uL	3/14 (21.4)	2/7 (28.6)	1.0000
HGB < 12.0 g/dL	6/14 (42.9)	1/7 (14.3)	0.3371
PLT < 150 K/uL	2/14 (14.3)	1/7 (14.3)	1.0000
Absolute neutrophils < 1.8 K/uL	2/14 (14.3)	0/7 (0)	0.5333
Absolute lymphocytes < 1.2 K/uL	3/14 (21.4)	2/7 (28.6)	1.0000
Positive ESR > 19 mm/h	3/12 (25)	2/6 (33.3)	1.0000
Positive CRP > 8.0 mg/L	6/12 (50)	2/7 (28.6)	0.6332
Total IgG > 1295 mg/dL	5/13 (33.3)	0/6 (0)	0.1280
C3 < 81 mg/dL	1/11 (9)	0/7 (0)	1.0000
C4 < 12 mg/dL	1/13 (7.7)	0/7 (0)	1.0000
Positive RF > 14 IU/mL	6/13 (46.2)	3/7 (42.9)	1.0000
Free K:L > 1.7	2/12 (16.7)	0/6 (0)	0.5294

Percentages of subjects with abnormal results are listed. *P*-values listed were calculated by Fisher’s test; significant *p*-values comparing percentages are labeled as follows: *p < 0.05, ***p < 0.001. ANA, antinuclear antibody; WBC, white blood cells; HGB, hemoglobin; PLT, platelets; ESR, sedimentation rate; CRP, C-reactive protein; IgG, immunoglobulin G; C3, complement 3; C4, complement 4; RF, rheumatoid factor; Free K:L, free kappa light chain to free lambda light chain ratio.

**FIGURE 4:**
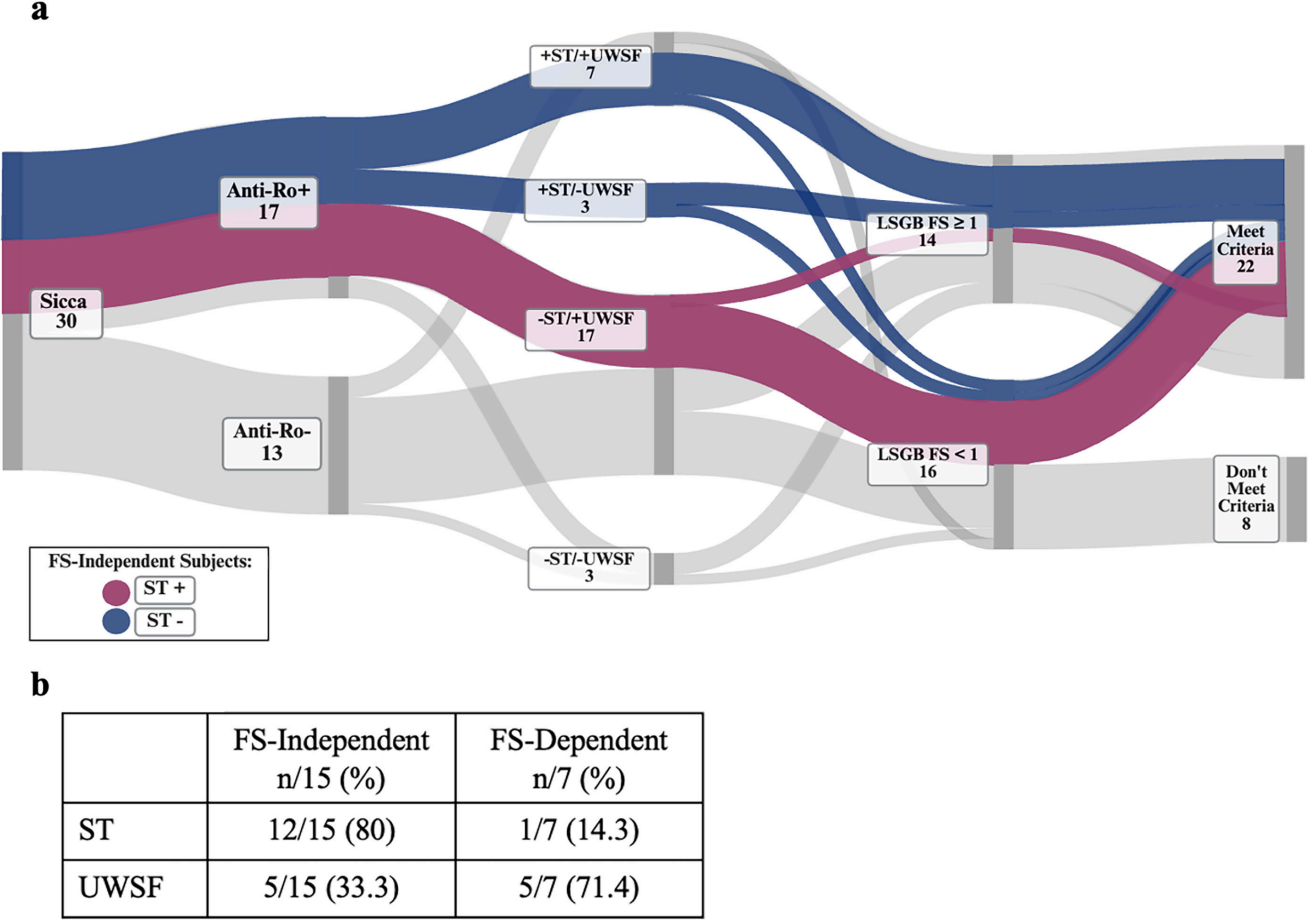
Concordance of ST and LSGB results in FS-independent subjects that meet SjD classification. **(a)** Visualization of serologic, histopathologic, and contemporaneous oral/ocular dryness testing of subjects projected in Sankey plot. FS-independent pathways to meeting ACR/EULAR SjD classification criteria are highlighted according to ST result. **(b)** Percentages of LSGB FS results concordant with ST or UWSF results are listed. Positive ST (purple), negative ST (blue).

We visualized serologic, histopathologic, and oral/ocular dryness testing results in a Sankey plot, highlighting FS-independent pathways according to ST positivity or negativity. We observed that 6/8 (75%) FS-independent subjects with positive ST had positive FS (blue pathway), and 6/7 (86%) FS-independent subjects with negative ST had a negative FS (purple pathway) (Figure 4a). Thus, 12/15 (80%) FS-independent subjects exhibited concordance between LSGB and ST results, while only 1/7 (14.3%) FS-dependent subject exhibited concordance. We did not observe such a pattern with UWSF (Figure 4b).

## 4 Discussion

The clinical heterogeneity of SjD, encompassing both glandular and extraglandular manifestations, complicates diagnosis, particularly in patients with atypical presentations. While multidisciplinary evaluation is often necessary–especially in seronegative cases requiring histopathologic confirmation via LSGB–our data demonstrate that LSGB provides modest incremental diagnostic value in seropositive SjD. However, our data do not subvert its significant clinical value, particularly in lymphoma risk calculation for SjD (9). Most seropositive patients met classification criteria through objective oral/ocular dryness measures, independent of LSGB results. This underscores the utility of integrating these non-invasive tests into routine workflows to reduce biopsy dependence for meeting classification criteria and to evaluate SjD cases comprehensively.

A key limitation of LSGB lies in its technical variability, which complicates interpretation. Challenges include inconsistent glandular sampling, ambiguous reporting of non-specific chronic sialadenitis, and discrepancies in FS calculation due to ductal atrophy, fibrosis, or inter-observer variability (10). These issues are particularly problematic in late-stage SjD, where fibrotic or atrophied glands may yield falsely low FS values, risking misclassification. In contrast, contemporaneous ST and UWSF–central to the 2016 ACR/EULAR criteria–provide standardized, functional assessments of dryness that circumvent these histopathologic pitfalls.

While classification criteria are not synonymous with clinical diagnosis, (11) they offer a structured framework to address diagnostic complexity in SjD. For instance, objective dryness may not be evident in SjD in earlier stages of disease, though may be able to still capture subclinical dysfunction (12). Objective dryness metrics not only quantify disease severity but also bridge the well-documented discordance between subjective symptoms and objective findings (13). For instance, our cohort included patients with profound ocular damage who lacked subjective dryness complaints, echoing prior reports of asymptomatic disease. Furthermore, 53% of FS-independent subjects in our study would have failed classification criteria without ST or UWSF testing, highlighting the indispensability of these tests in seropositive patients with equivocal or negative biopsies.

Notably, ST exhibited stronger correlations with LSGB positivity that were not evident with UWSF. The inverse relationship between ST values and FS, along with 80% concordance between positive ST and LSGB, suggests ST aligns with LSGB and disease activity in select cases. Conversely, the fact that low UWSF did not have an association with abnormal FS aligns with mixed findings in prior studies, (14–16) suggesting its diagnostic performance may require refinement. Nevertheless, UWSF remains valuable for objectively capturing oral dryness severity.

Our clinical evaluation preserved LSGB accessibility but in this retrospective analysis, we prioritized evaluating the added diagnostic utility of dryness testing. Only 11.8% of seropositive subjects required LSGB for classification, reinforcing that most seropositive SjD cases can be diagnosed without biopsy when ST and UWSF are utilized. Even in this minority, dryness testing remained valuable by revealing a distinct subgroup without objective sicca, thereby underscoring disease heterogeneity and offering potential insight into disease stage (17). Despite barriers such as the non-billable status of dryness testing in the U.S.–a likely contributor to its underuse–our findings advocate for its integration into clinical practice. These tests provide timely, actionable insights that enhance diagnostic accuracy while reducing reliance on invasive biopsies for meeting classification criteria, particularly in seropositive populations.

This analysis has several limitations. First, the study’s limited cohort size may restrict the generalizability of findings, particularly given the heterogeneity inherent to SjD phenotypes. Second, the absence of ocular staining scores–a component of the ACR/EULAR criteria–likely influenced the observed reliance on ST and UWSF rates as surrogate dryness measures. This omission precludes definitive conclusions about whether a fully non-invasive diagnostic pathway (e.g., serology, ocular staining, UWSF, and ST) could complement LSGB in borderline cases. For example, the 2/17 anti-Ro-positive subjects who required LSGB for classification despite negative ST/UWSF results might still meet criteria non-invasively if ocular staining data were available, underscoring the need for standardized multimodal dryness assessments. Prospective studies with larger cohorts should prioritize integrating ocular staining scores to validate the robustness of non-invasive classification pathways. Additionally, longitudinal evaluation of oral/ocular dryness metrics could clarify their temporal stability and prognostic utility, particularly in seropositive patients with fluctuating symptom severity.

## 5 Conclusion

Given the underutilization of dryness testing in real-world rheumatologic evaluations for suspected SjD, we aimed to assess the added value of non-invasive ocular and oral dryness tests relative to invasive LSGB. Our findings show that approximately 81% of subjects meeting SjD classification criteria did so based solely on non-invasive testing (anti-Ro positivity and ST and/or UWSF). A significant correlation was observed between abnormal ST and LSGB FS ≥ 1, though this pattern was not evident with LSGB and anti-Ro serology or UWSF. These results suggest that contemporaneous dryness testing should be integrated into routine rheumatologic care, as many suspected SjD cases meet classification criteria independent of LSGB histopathology.

## Data Availability

The raw data supporting the conclusions of this article will be made available by the authors, without undue reservation.
